# Prevalence and factors associated with anxiety and depression among Chinese prison officers during the prolonged COVID-19 pandemic

**DOI:** 10.3389/fpubh.2023.1218825

**Published:** 2023-08-03

**Authors:** Yuze Zeng, Junze Xiao, Qingqi Zhang, Xiaoqian Liu, Ai Ma

**Affiliations:** ^1^School of Criminal Justice, China University of Political Science and Law, Beijing, China; ^2^School of Sociology, China University of Political Science and Law, Beijing, China

**Keywords:** COVID-19, prison officer, anxiety, depression, risk factors, protective factors

## Abstract

**Objective:**

This study examined the prevalence of anxiety and depression—along with the potential risk and protective factors—among Chinese prison officers during the prolonged COVID-19 pandemic.

**Method:**

A cross-sectional survey of 1,268 officers from five prisons in western and southern China was administered between June and July 2022. The questionnaires comprised two sections. In the first section, the Generalized Anxiety Disorder-7 (GAD-7) and Patient Health Questionnaire-9 (PHQ-9) were used to evaluate the prevalence of anxiety and depression, respectively, among prison officers. In the second section, the potential influencing factors were examined. Categorical data were compared using χ^2^ tests and *t*-tests; binary logistic regression analysis was performed to identify factors associated with anxiety and depression.

**Results:**

The prevalence rates of anxiety and depression among the prison officers were 72.6% and 69.8%, respectively. Risk factors for anxiety were older age, being unmarried, work–family conflicts, job demands, and COVID-19 burnout; protective factors were exercise, positive family relationships, and group cohesion. Work–family conflicts, job demands, intolerance of uncertainty regarding COVID-19, and COVID-19 burnout were risk factors for depression, whereas annual income >150,000 RMB, exercise, positive family relationships, group cohesion, and job autonomy were protective factors against depression.

**Conclusion:**

The prevalence of anxiety and depression among Chinese prison officers was relatively high during the prolonged COVID-19 pandemic, and more targeted measures should be implemented to improve their mental health. This study offers a reference for improving prison officers’ mental health in response to similar public health emergencies in the future.

## 1. Introduction

Since the end of 2019, COVID-19 has been spreading rapidly worldwide (1). The World Health Organization’s Emergency Committee declared a global public health emergency on January 30, 2020 (2). Globally, the COVID-19 pandemic and measures implemented to protect and prevent its spread have exerted adverse effects on the mental health of various populations, including the general population as well as specific subgroups, such as students, pregnant women, and healthcare workers (3). In China, the government instituted numerous efficacious management strategies to mitigate this public health crisis. Considering the challenges associated with prison environments, including overcrowding, inadequate ventilation, and numerous prisoners with underlying health problems, the prison system instituted a prolonged closed-loop management approach referred to as the “lockdown shifts” strategy. Nearly three years have elapsed since the implementation of the “lockdown shifts” strategy in response to the COVID-19 outbreak at the end of 2019. This strategy requires prison officers to be divided into three groups for shifts. Each group undergoes three stages in a cycle—specifically, isolation at the designated place, lockdown shifts in the prison, and a rest period at home. Each stage lasts a minimum of 7 days, with specific requirements adjusted according to the local COVID-19 situation (4).

Compared to prisons in other nations severely influenced by the COVID-19 pandemic (5–8), China’s preventative measures have proven capable of curbing the virus’ proliferation within the jail system. Nevertheless, COVID-19’s prolonged prevalence has substantially burdened prison officers, thereby disrupting their work-life balance (4). Prior research has indicated that doctors, civil servants, and police officers—working on the front lines of combating the COVID-19 pandemic—all experience mental health problems, predominantly anxiety and depressive symptoms (9–11). Therefore, assuming that prison officers in China face similar mental health challenges is reasonable. First, to avoid COVID-19’s spread in prisons, prison officers must experience a long period of isolation before entering the workspace. Previous studies have demonstrated that isolation measures increase prevalence rates of anxiety and depression directly (12, 13), and data from a survey of the prison staff in Turkey support this finding (14). Additionally, prison officers’ prolonged separation from their families during this period may contribute to loneliness, work fatigue, and work–family conflict, which have been linked with high levels of anxiety and depression (15–17). Furthermore, COVID-19 prevention and control measures have intensified prison officers’ workload, with the “lockdown shifts” strategy reducing the available workforce, thereby increasing job demands and exacerbating occupational stress. Ample research has indicated that occupational stress causes various mental health problems (18). Finally, isolation often results in the adoption of unhealthy lifestyles, such as irregular diet and lack of physical exercise (19, 20), which also pose threats to individuals’ mental health status. Generally, anxiety and depression are the two most common symptoms among prison officers that not only cause serious damage to their work efficacy (21), but are also associated with an increased risk of suicidal ideation and behavior (22, 23). Thus, an investigation focusing on prison officers’ anxiety and depression status after the “lockdown shifts” strategy’s implementation is urgently needed. Such a study could provide a reference to develop more precise and effective mental health intervention measures for prison officers.

During the COVID-19 outbreak’s initial stages, several studies investigated prison officers’ mental health status. Li et al. employed the 12-item General Health Questionnaire (GHQ-12) to survey Western Chinese frontline prison officers, finding that mental health issues’ prevalence was 33.43% (24). According to data from US correctional facilities, approximately 32% of correctional officers reported mild-to-severe depression symptoms, while 38% expressed mild-to-severe anxiety symptoms (25). The anxiety and depression rates among Turkish jail officers working under compulsory COVID-19 isolation measures were even more severe, with 48.9% exhibiting anxiety symptoms and 92.9% presenting depressive symptoms (14). However, these studies focused on psychopathological symptoms’ prevalence in prison officers at the pandemic’s commencement. With the virus continued spread, prison officers have experienced prolonged exposure to a stressful occupational situation. To date, few studies have explored the mental health status of prison officers during the prolonged COVID-19 pandemic, especially in the context of the long-term implementation of the “lockdown shifts” strategy, which is the primary focus of this study. Previous studies discovered that long-term exposure to a stressful occupational environment could have a greater detrimental effect on mental health (26, 27); further, reportedly, unresolved chronic stressors decrease positive emotions and increase negative sentiment, which are vulnerability factors for severe anxiety and depression symptoms (28). Moreover, neurobiological evidence has indicated that persistent stress from COVID-19 may chronically expose individuals to high cortisol levels, which stimulate the mesolimbic reward pathway in the brain, precipitating numerous health problems (29). However, prior studies have presented inconsistent results, with some indicating that upon experiencing prolonged stress, individuals can adapt to stressors and restore their mental health with the assistance of psychological resilience (30, 31). According to a survey administered in the United Kingdom, during the COVID-19 pandemic, individuals’ mental health initially deteriorated, followed by stabilization as the pandemic persisted (32). Thus, prison officers’ mental health status with the COVID-19 pandemic’s prolongation—compared to that at the outbreak’s beginning—remains uncertain and must be further investigated.

**Figure 1:**
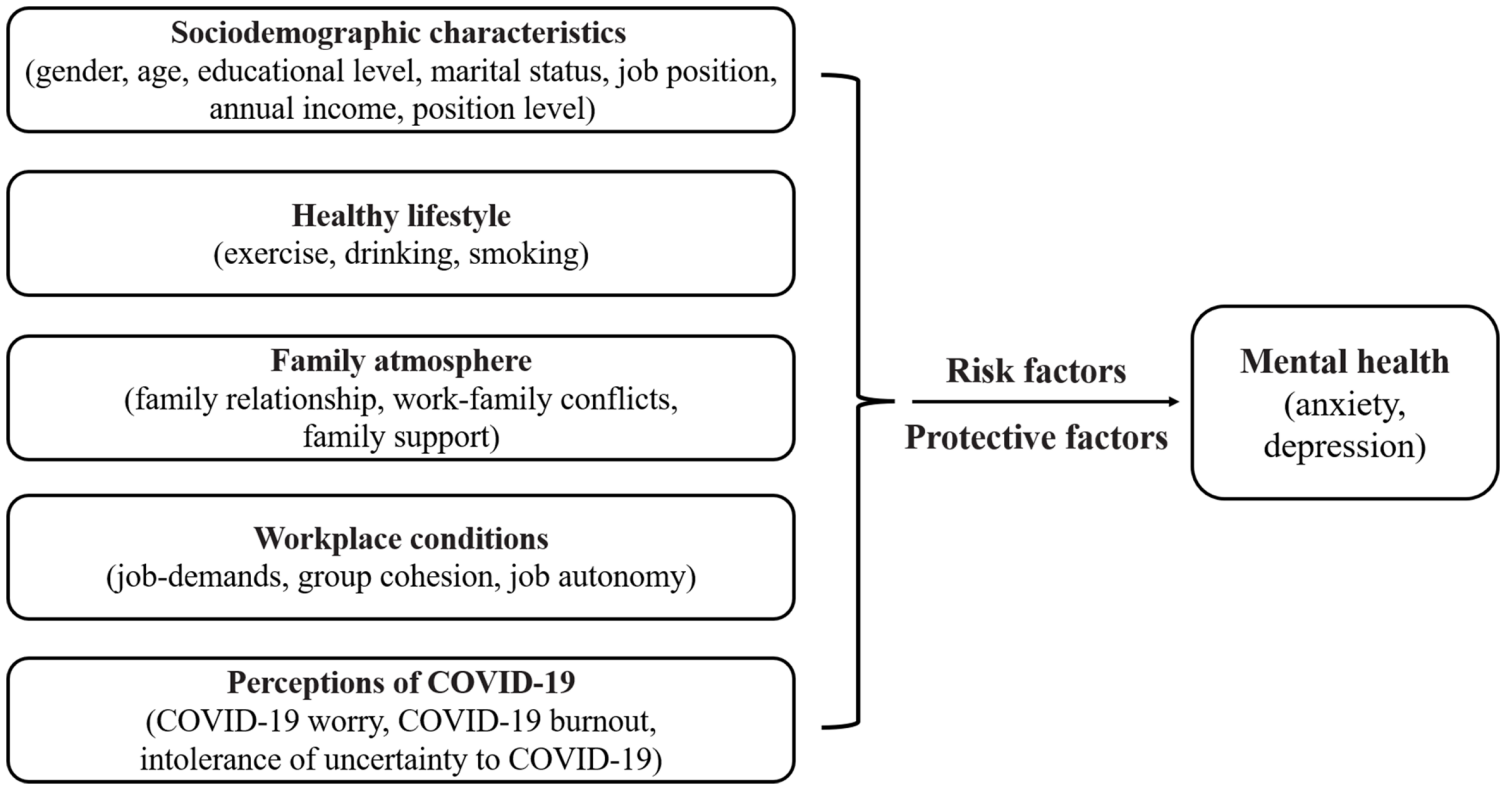
Study framework.

This study is the first to investigate anxiety and depression among prison officers during the extended COVID-19 pandemic. Per the stress and coping transactional model, an individual’s psychological responses to stressors depend on their cognitive appraisal, coping resources, and personal characteristics (33). The COVID-19 pandemic has exerted a sustained negative impact on prison officers’ lives, thus acting as a chronic stressor (34). Their perception of COVID-19—as a cognitive appraisal—can influence their selection of coping strategies, potentially exacerbating mental health issues. Sociodemographic factors, including age, gender, and educational level, shape individuals’ cognitive appraisal and coping resources when facing stress (35). Moreover, personal habits, including healthy eating and exercise, are linked to adaptability and coping strategies (36). Furthermore, family support, coworker relationships, and job-related stress in the work environment affect the availability of coping resources and choices (37). Based on this model, and considering prison officers’ unique living and working situations during the COVID-19 pandemic, we propose a research framework that includes five factors—namely, sociodemographic characteristics, healthy lifestyle, family environment, workplace conditions, and COVID-19 perceptions (Figure 1). This study examined the prevalence of anxiety and depression—along with the potential risk and protective factors involved—among prison officers over a prolonged period. As of January 8, 2023, China managed COVID-19 as a “Category B” infectious disease, thus marking the termination of the prison “lockdown shifts” strategy; nevertheless, the global pandemic and evolving virus strains persist. Our findings can aid prison systems in protecting officers’ mental health during future emergencies that necessitate closed-loop management’s adoption.

## 2. Methods

### 2.1. Study design, participants, and procedure

This cross-sectional study was conducted in five prisons in Western and Southern China between June and July 2022. Noteworthily, owing to the government’s diligent efforts, the pandemic’s spread in the regions where the five prisons are situated was effectively controlled during the investigation period, resulting in only a few reported cases of infection. This study distributed questionnaires to 1,328 prison officers through a most widely used online survey platform in China,^1^ and established three exclusion criteria listed below: (1) questionnaire that provided evident incorrect information; (2) questionnaire that was completed within 3 min; and (3) questionnaire wherein participant consistently selected the same option across a substantial portion of the questionnaire. Satisfying any one of the three exclusion criteria renders the survey invalid. After excluding 60 invalid questionnaires, the final sample comprised 1,268 prison officers (735 men and 533 women). In terms of job position, there were 778 frontline and 490 non-frontline prison officers. The data were anonymously collected, and data confidentiality was ensured. All participants completed the questionnaire after signing informed consent forms.

### 2.2. Measurements

A two-section questionnaire was administered. The first section evaluated participants’ anxiety and depression status. The second section measured factors that may affect their anxiety and depression status.

#### 2.2.1. Section 1

The General Anxiety Disorder-7 (GAD-7) and Patient Health Questionnaire 9 (PHQ-9) were used to assess the level of anxiety and depression, respectively. The GAD-7 contains seven items, each of which is scored on a four-point Likert scale ranging from 0 (none) to 3 (nearly every day). Total scores of 0–4, 5–9, 10–14, and 15–21 were categorized as minimal, mild, moderate, and severe symptoms of anxiety, respectively (38). In this study, Cronbach’s *α* for the GAD-7 was 0.963. The PHQ-9 contains nine items, each of which is scored using a four-point Likert scale (0 = none to 3 = nearly every day). Total scores of 0–4, 5–9, 10–14, 15–19, and 20–27 indicate minimal, mild, moderate, moderately severe, and severe depression symptoms, respectively (39). In this study, Cronbach’s α for the PHQ-9 was 0.947. A total score of ≥5 was adopted to identify individuals with the presence of mild to severe anxiety or depressive symptoms (40, 41).

#### 2.2.2. Section 2

##### 2.2.2.1. Sociodemographic characteristics

Participants’ sociodemographic characteristics—namely, gender, age, educational level, marital status, annual income, job position, and position level were collected.

##### 2.2.2.2. Healthy lifestyle

Healthy lifestyle refers to changes in participants’ habits—including smoking, drinking, and physical exercise—during the prolonged COVID-19 pandemic. These habits were assessed via three items—specifically, “During the COVID-19 pandemic, have there been any changes in your smoking habits?” “During the COVID-19 pandemic, have there been any changes in your drinking habits?”, and “During the COVID-19 pandemic, have there been any changes in your physical exercise habits?” Responses were scored on a five-point Likert-type scale ranging from 1 (significantly decreased) to 5 (significantly increased).

##### 2.2.2.3. Family environment

Family environment information was evaluated in the COVID-19 pandemic’s context—namely, family relationships’ quality, work–family conflict, and family support. Family relationships’ quality was measured by asking, “How is the quality of your family relationships?” Responses ranged from 1 (extremely bad) to 5 (extremely good). Netemeyer et al. (42) Work–Family Conflict Scale was used to measure the level of work–family conflict, comprising five items, such as “The amount of time my job takes up makes it difficult to fulfill family responsibilities,” with responses ranging from 1 = never to 5 = always. This scale exhibited an acceptable Cronbach’s alpha value of 0.872. Family support—the level of support provided by family with respect to work—was measured using the four-item scale developed by Ellison (43). An example item is “I can turn to my family when my job upsets me,” with responses ranging from 1 = completely disagree to 4 = completely agree. The Cronbach’s alpha value for the four items was 0.760.

##### 2.2.2.4. Workplace conditions

Workplace conditions include job demands, job autonomy, and group cohesion. The questionnaire on job demands includes two dimensions—namely, quantitative and emotional. Quantitative requirements were assessed based on the job content instrument developed by Karasek (44), which measures quantitative workload and time pressure via five items. Emotional requirements were assessed using the Emotional Requirement Subscale of the Work Experience and Evaluation Questionnaire developed by Van Veldhoven and Meijman (45), which comprises six items that quantify emotional strain experienced in the work context (46). The Cronbach’s alpha value for the eleven items was 0.878. Job autonomy was measured using the Job Content Instrument, which includes three items (43) that probe workers’ sense of autonomy in the workplace. An example item is “Most of the time, I am able to choose how my tasks are completed during my shift,” with responses ranging from 1 = completely to 5 = not at all. The Cronbach’s alpha value of the three items was 0.806. Group cohesion was measured using a revised version of the Group Cohesion Scale developed by Dobbins and Zaccaro (47). Eight items—such as “My colleagues in prison get along very well”—were assessed, rated from 1 (strongly disagree) to 7 (strongly agree). In this study, the Group Cohesion Scale exhibited a Cronbach’s *α* of 0.787.

##### 2.2.2.5. Perceptions of COVID-19

Perceptions of COVID-19 include COVID-19 burnout, COVID-19 worry, and intolerance of uncertainty regarding COVID-19. The COVID-19 Burnout Scale developed by Yildirim and Solmaz was used to measure the degree of COVID-19 burnout. It comprises 10 items, including “When you think about COVID-19 overall, how often do you feel hopeless?” (1 = never to 5 = always) (48). This scale exhibited a Cronbach’s *α* of 0.969. Intolerance of uncertainty regarding COVID-19 was measured using a scale adapted by Dai et al. (49) based on the Intolerance of Uncertainty Scale. Three items were assessed, including “The uncertainty of the COVID-19 pandemic has seriously affected my life” (1 = strongly disagree to 7 = strongly agree). The Cronbach’s alpha value of the three items was 0.777. COVID-19 worry was measured using a self-developed instrument comprising the following three questions: “Are you worried about yourself or your family or friends being infected with COVID-19?” “Are you worried about COVID-19 spread in prison?” and “Are you worried about people around you being infected with COVID-19?” (1 = yes, 2 = no).

### 2.3. Statistical analysis

For data processing, SPSS 26.0 was used. First, a descriptive statistical analysis of the demographic characteristics was conducted. Subsequently, bivariate analysis was used for categorical comparisons, which were described as numbers and proportions (%). The chi-square test was performed to compare the proportions of participants reporting anxiety or depression. Normally distributed continuous variables were summarized as mean ± SD and compared using a t-test. The relationships between the dependent variables and potential risk or protective factors were examined using binary logistic regression analysis. In the logistic regression model, only variables with a bivariate *p*-value less than 0.1 were included. The regression analysis’ results are presented as odds ratios (ORs) and confidence intervals (CIs) with a confidence level of 95%.

## 3. Results

### 3.1. Sociodemographic characteristics of participants

Table 1 presents the 1,268 prison officers’ sociodemographic characteristics. Overall, 58.0% of the participants were men and 42% were women, and approximately half (51.8%) of them were aged from 30 to 49. Most participants (78.5%) had a bachelor’s degree, and over four-fifth (82.3%) of the sample was married; further, 61.4% and 38.6% of participants were frontline and non-frontline prison officers, respectively. The majority had an annual income of 100,000 to 150,000 RMB. Most (78.7%) prison officers held a non-leader position.

**Table 1:** Prison officers’ sociodemographic characteristics.

Sociodemographic characteristics	(*N* = 1,268) *n* (%)
Gender	
Men	735 (58)
Women	533 (42)
Age	
20–29	179 (14.1)
30–39	389 (30.7)
40–49	267 (21.1)
≥50	433 (34.1)
Educational level	
Below associate degree	26 (2.1)
Associate degree	229 (18.1)
Bachelor’s degree	996 (78.5)
Master’s degree or above	17 (1.3)
Marital status	
Married	1,044 (82.3)
Single	155 (12.2)
Divorced (or widowed)	69 (5.5)
Job position	
Frontline	778 (61.4)
Non-frontline	490 (38.6)
Annual income	
<100,000 RMB	100 (7.9)
100,000–150,000 RMB	954 (75.2)
>150,000 RMB	214 (16.9)
Position level	
Department-level leader	69 (5.4)
Section-level leader	201 (15.9)
Non-leader	998 (78.7)

One RMB is equivalent to 0.13 US Dollar or 0.14 Euro.

### 3.2. Prevalence of anxiety and depression

Anxiety symptoms were reported by 69.8% (885/1268) of the prison officers, whereas 72.6% (920/1268) reported depressive symptoms. Bivariate analysis revealed that anxiety was significantly associated with age, marital status, job position, annual income, position level, exercise, alcohol consumption, smoking, family relationships, work–family conflict, family support, job demands, group cohesion, job autonomy, COVID-19 burnout, and intolerance of uncertainty regarding COVID-19 (*p* < 0.05, Table 2). As presented in Table 3, bivariate analyses of depression and anxiety revealed generally similar findings.

**Table 2:** Prison officers’ characteristics categorized into anxiety and non-anxiety groups.

		Non-anxiety	Anxiety			
Variables	Categories	*n* (%) or m ± SD		χ^2^ or t	*p*-value	*φ* or *Cohen’s d*
**Sociodemographics**						
Gender	Men	224 (30.5)	511 (69.5)	0.061	0.805	0.007
	Women	159 (29.8)	374 (70.2)			
Age	20–29	49 (27.4)	130 (72.6)	24.115	<0.001	0.138
	30–39	88 (22.6)	301 (77.4)			
	40–49	81 (30.3)	186 (69.7)			
	≥50	165 (38.1)	268 (61.9)			
Educational level	Below associate degree	7 (26.9)	19 (73.1)	0.662	0.882	0.023
	Associate degree	72 (31.4)	157 (68.6)			
	Bachelor	300 (30.1)	696 (69.9)			
	Master or above	4 (23.5)	13 (76.5)			
Marital status	Married	330(31.6)	714(68.4)	7.698	0.021	0.078
	Unmarried	32 (20.6)	123 (79.4)			
	Divorced (or widowed)	21 (30.4)	48 (69.6)			
Job position	Frontline	175 (22.5)	603 (77.5)	56.790	<0.001	0.212
	Non-frontline	208 (42.4)	282 (57.6)			
Annual income (RMB)	<100,000	25(25.0)	75(75.0)	23.295	<0.001	0.136
	100,000–150,000	264 (27.7)	690 (72.3)			
	>150,000	94 (43.9)	120 (56.1)			
Position level	Department-level leader	35 (50.7)	34 (49.3)	15.190	0.001	0.109
	Section-level leader	63 (31.3)	138 (68.7)			
	Non-leader	285 (28.6)	713 (71.4)			
**Healthy lifestyle**						
Exercise		3.01 ± 1.17	2.67 ± 1.34	4.349	<0.001	0.263
Drink alcohol		2.75 ± 0.95	3.04 ± 1.01	−4.884	<0.001	0.292
Smoke		2.99 ± 0.55	3.18 ± 0.76	−4.252	<0.001	0.270
**Family environment**						
Family relationship		4.49 ± 0.66	4.18 ± 0.79	6.597	<0.001	0.412
Work–family conflict		26.26 ± 7.40	33.15 ± 7.03	−15.764	<0.001	0.964
Family support		11.22 ± 2.09	10.43 ± 2.31	5.798	<0.001	0.352
**Workplace conditions**						
Job demands		37.14 ± 7.29	43.55 ± 6.89	−14.935	<0.001	0.914
Job autonomy		10.03 ± 2.76	8.34 ± 2.99	9.443	<0.001	0.578
Group cohesion		46.09 ± 7.25	41.09 ± 8.00	10.501	<0.001	0.643
**Perceptions of COVID-19**						
COVID-19 worry		4.55 ± 0.84	4.48 ± 0.81	1.387	0.166	0.085
COVID-19 burnout		17.93 ± 7.72	29.58 ± 10.83	−19.059	<0.001	1.167
Intolerance of uncertainty regarding COVID-19		14.87 ± 4.11	17.32 ± 3.42	−10.982	<0.001	0.673

**Table 3:** Prison officers’ characteristics categorized into depression and non-depression groups.

		Non-depression	Depression			
Variables	Categories	*n* (%) or m ± SD		χ^2^ or *t*	*p*-value	*φ* or *Cohen’s d*
**Sociodemographics**						
Gender	Men	204 (27.8)	531 (72.2)	0.085	0.771	0.008
	Women	144 (27.0)	389 (73.0)			
Age	20–29	38 (21.2)	141 (78.2)	31.186	<0.001	0.157
	30–39	77 (19.8)	312 (80.2)			
	40–49	77 (28.8)	190 (71.2)			
	≥50	156 (36.0)	277 (64.0)			
Educational level	Below associate degree	9 (34.6)	17 (65.4)	2.524	0.471	0.045
	Associate degree	69 (30.1)	160 (69.9)			
	Bachelor’s	267 (26.8)	729 (73.2)			
	Master’s or above	3 (17.6)	14 (82.4)			
Marital status	Married	304 (29.1)	740 (70.9)	9.566	0.008	0.087
	Unmarried	27 (17.4)	128 (82.6)			
	Divorced (or widowed)	17 (24.6)	52 (75.4)			
Job position	Frontline	157 (20.2)	621 (79.8)	53.361	<0.001	0.205
	Non-frontline	191 (39.0)	299 (61.0)			
Annual income (RMB)	<100,000	16(16.0)	84(84.0)	49.004	<0.001	0.197
	100,000–150,000	233 (24.4)	721 (75.6)			
	>150,000	99 (46.3)	115 (53.7)			
Position level	Department-level leader	37 (53.6)	32 (46.4)	35.795	<0.001	0.168
	Section-level leader	71 (35.3)	130 (64.7)			
	Non-leader	240 (24.0)	758 (76.0)			
**Healthy lifestyle**						
Exercise		3.07 ± 1.21	2.66 ± 1.32	5.124	<0.001	0.318
Drink alcohol		2.74 ± 0.92	3.03 ± 1.02	−4.616	<0.001	0.292
Smoke		3.00 ± 0.56	3.16 ± 0.75	−3.635	<0.001	0.227
**Family environment**						
Family relationship		4.51 ± 0.64	4.19 ± 0.79	6.760	<0.001	0.427
Work–family conflict		25.98 ± 7.07	33.00 ± 7.19	−15.588	<0.001	0.981
Family support		11.32 ± 2.09	10.43 ± 2.29	6.330	<0.001	0.398
**Workplace conditions**						
Job demands		37.32 ± 7.06	43.23 ± 7.16	−13.184	<0.001	0.829
Job autonomy		10.21 ± 2.65	8.34 ± 2.99	10.269	<0.001	0.645
Group cohesion		46.22 ± 7.08	41.24 ± 8.06	10.148	<0.001	0.638
**Perceptions of COVID-19**						
COVID-19 worry		4.54 ± 0.83	4.49 ± 0.82	0.934	0.351	0.061
COVID-19 burnout		17.46 ± 7.50	29.31 ± 10.83	−18.781	<0.001	1.182
Intolerance of uncertainty regarding COVID-19		14.69 ± 4.17	17.30 ± 3.40	−11.440	<0.001	0.720

### 3.3. Risk and protective factors for anxiety and depression

We examined prison officers’ risk and protective factors for anxiety and depression using binary logistic regression. Collinearity diagnostics were conducted for all factors involved in binary logistic regression (Table 4), which indicated that all factors’ tolerances were greater than 0.5, and that all factors’ VIF values are less than 2, suggesting that no significant collinearity exists herein.

**Table 4:** Factors related to anxiety and depression according to the binary logistic regression model.

Variables	Collinearity diagnostics		Factors	Anxiety		Depression	
	Tolerance	VIF		OR (95% CI)	*p*-value	OR (95% CI)	*p*-value
**Sociodemographics**							
Age	0.681	1.468	20–29	[Reference]		[Reference]	
			30–39	2.268 (1.204–4.274)	0.011	2.063 (1.053–4.041)	0.035
			40–49	3.172 (1.573–6.396)	0.001	2.558 (1.227–5.333)	0.012
			≥50	3.683 (1.883–7.203)	<0.001	2.731 (1.354–5.511)	0.005
Marital status	0.889	1.124	Married	[Reference]		[Reference]	
			Unmarried	2.880 (1.447–5.731)	0.003	1.990 (0.971–4.079)	0.060
			Divorced (or widowed)	0.897 (0.435–1.848)	0.768	1.294 (0.598–2.798)	0.513
Job position	0.694	1.440	Frontline	[Reference]		[Reference]	
			Non-frontline	0.968 (0.675–1.389)	0.860	1.218 (0.838–1.772)	0.301
Annual income (RMB)	0.693	1.443	<100,000	1[Reference]		1[Reference]	
			100,000–150,000	1.021 (0.523–1.994)	0.952	0.581 (0.275–1.230)	0.156
			>150,000	0.716 (0.322–1.590)	0.411	0.312 (0.131–0.743)	0.009
Position level	0.735	1.360	Department-level leader	[Reference]		[Reference]	
			Section-level leader	0.864 (0.404–1.851)	0.708	0.660 (0.305–1.428)	0.292
			Non-leader	0.772 (0.380–1.569)	0.474	1.071 (0.525–2.187)	0.850
**Healthy lifestyle**							
Exercise	0.953	1.049		0.867 (0.767–0.980)	0.023	0.813 (0.716–0.924)	0.002
Drink alcohol	0.831	1.203		1.157 (0.974–1.375)	0.097	1.096 (0.919–1.308)	0.307
Smoke	0.852	1.174		1.143 (0.894–1.462)	0.286	1.050 (0.818–1.350)	0.700
**Family environment**							
Family relationship	0.858	1.166		0.738 (0.586–0.930)	0.010	0.748 (0.589–0.950)	0.017
Work–family conflicts	0.525	1.906		1.037 (1.009–1.065)	0.009	1.043 (1.014–1.072)	0.003
Family support	0.806	1.240		0.983 (0.907–1.066)	0.683	0.953 (0.877–1.036)	0.262
**Workplace conditions**							
Job demands	0.618	1.619		1.087 (1.058–1.116)	<0.001	1.066 (1.038–1.095)	<0.001
Job autonomy	0.745	1.343		0.958 (0.901–1.020)	0.179	0.931 (0.873–0.992)	0.027
Group cohesion	0.784	1.275		0.950 (0.928–0.972)	<0.001	0.961 (0.939–0.984)	0.001
**Perceptions of COVID-19**							
COVID-19 burnout	0.601	1.664		1.101 (1.078–1.123)	<0.001	1.102 (1.079–1.127)	<0.001
Intolerance of uncertainty regarding COVID-19	0.709	1.410		1.036 (0.989–1.085)	0.132	1.059 (1.009–1.110)	0.019

As presented in Table 4, the results indicated that risk factors for anxiety among prison officers were older age, being unmarried, work–family conflicts, job demands, and COVID-19 burnout, while protective factors were exercise, family relationships, and group cohesion. Risk factors for depressive symptoms were older age, work–family conflicts, job demands, intolerance of uncertainty regarding COVID-19, and COVID-19 burnout, while protective factors were annual income of greater than 150,000 RMB, exercise, family relationships, group cohesion, and job autonomy. Specifically, compared to prison officers aged 20–29, those aged 30–39 (OR = 2.268; 95% CI = 1.204–4.274), 40–49 (OR = 3.172; 95% CI = 1.573–6.396), and ≥ 50 (OR = 3.683; 95% CI = 1.883–7.203) were more likely to experience anxiety symptoms. Compared to married prison officers, unmarried prison officers (OR = 2.880; 95% CI = 1.447–5.731) exhibited a significantly higher probability of experiencing anxiety symptoms. Prison officers with more severe work–family conflicts (OR = 1.037; 95% CI = 1.009–1.065), higher job demands (OR = 1.087; 95% CI = 1.058–1.116), and COVID-19 burnout (OR = 1.101; 95% CI = 1.078–1.123) were more likely to experience anxiety. Those who exercised regularly (OR = 0.867; 95% CI = 0.767–0.980), had desirable family relationships (OR = 0.738; 95% CI = 0.586–0.930), and group cohesion (OR = 0.950; 95% CI = 0.928–0.972) were less likely to present anxiety symptoms. Compared to prison officers aged 20–29, those aged 30–39 (OR = 2.063; 95% CI = 1.053–4.041), 40–49 (OR = 2.558; 95% CI = 1.227–5.333), and ≥ 50 (OR = 2.731; 95% CI = 1.354 - 5.511) were more likely to experience depressive symptoms. Prison officers with severe work–family conflicts (OR = 1.043; 95% CI = 1.014–1.072), higher job demands (OR = 1.066; 95% CI = 1.038–1.095), COVID-19 burnout (OR = 1.102; 95% CI = 1.079–1.127), and intolerance of uncertainty regarding COVID-19 (OR = 1.059; 95% CI = 1.009–1.110) were more likely to experience depressive symptoms. Compared to prison officers with an annual income below 100,000 RMB, those with an annual income above 150,000 RMB (OR = 0.312; 95% CI = 0.131–0.743) were less likely to experience depressive symptoms. Prison officers who exercised regularly (OR = 0.813; 95% CI = 0.716–0.924), had desirable family relationships (OR = 0.748; 95% CI = 0.589–0.950), group cohesion (OR = 0.961; 95% CI = 0.939–0.984), and job autonomy (OR = 0.931; 95% CI =0.873–0.992) were less likely to develop depression.

## 4. Discussion

The COVID-19 virus’ worldwide spread has negatively impacted the mental health of various social communities—including frontline healthcare professionals, students, and civil servants (11, 50, 51). The Chinese prison system implemented a “lockdown shifts” strategy for nearly three years; this study was conducted before the prison system lifted this policy. We investigated the prevalence and factors associated with anxiety and depression in prison officers during the prolonged COVID-19 pandemic. Our study showed a prevalence of 69.8% and 72.6% of anxiety and depression, respectively, among Chinese prison officers.

For a long time, owing to prisons’ confined and tedious working environment, prison officers’ mental health challenges have received significant attention. A systematic review published in 2019—focusing on correctional officers—analyzed surveys from six countries, including China; the review found that the prevalence of anxiety and depression disorders in this group ranged from 12.2%–37.9% and 24%–59.7%, respectively (52). This systematic review included a study conducted by Carleton et al. (53) from 2016 to 2017, which used the same assessment tools as our study, and revealed that the mean scores of anxiety and depression among Canadian correctional officers were 6.08 and 7.33, respectively. Evidently, these scores were lower than the results obtained in our study (anxiety: 7.85, depression: 8.96). Thus, compared to the pre-COVID-19 stage, our findings might indicate a higher prevalence of anxiety and depressive symptoms among prison officers than previous studies. Meanwhile, compared to the research conducted at the COVID-19 outbreak’s onset, our study’s results are also significantly higher than those of prison officers in China (33.43%) and in the United States (38, 32%), as mentioned above (24, 25). Moreover, the mental health status of prison officers in this study was worse than that of other groups in China—including older adults, perinatal women, and medical residents (40, 41, 54)—who were also evaluated during the prolonged COVID-19 pandemic.

Notably, as the COVID-19 pandemic continues, China’s epidemic has proceeded from an initial severe outbreak to being effectively controlled at the time of the investigation. However, we observed an increasing prevalence of mental health issues among prison officers. This trend contrasts with those of studies conducted in medical staff, such as healthcare workers and nurses (55, 56), which have found that mental health problems were highly prevalent during the epidemic’s peak, but decreased as the outbreak subsided (57, 58). Potential reasons for this result may involve the comprehensive influence of various factors, including Chinese prison officers’ social background, culture, and occupational characteristics. On the one hand, China’s prison system follows a responsibility subcontracting system, whereby each prison officer is assigned specific tasks related to pandemic prevention and control, as well as security maintenance duties (4). These tasks are critical for evaluating their job performance. This increases work pressure for Chinese prison officers compared to other groups and prison officers in other countries. On the other hand, owing to prison officers’ unique occupational characteristics, their lives cannot improve and stabilize even after the pandemic is controlled. They are subject to closed-loop management measures for an extended period, including isolation and behavioral control. Consequently, they experience increased feelings of loneliness and worsening mental health (12, 59). Additionally, in Chinese culture, the concept of “face” holds great importance. Prison officers may be concerned regarding their occupational image and consider it shameful to ask for help, which makes them more likely to endure psychological problems rather than seek help (60). This cultural factor—compared to other countries—may make Chinese prison officers more vulnerable to mental health problems. Consequently, identifying the risk and protective factors affecting prison officers’ mental health, which can provide a reference for interventions for their mental health challenges, is imperative.

To this end, a binary logistic regression model was utilized. The results revealed that prison officers who were over the age of 30, were unmarried, experienced significant work–family conflicts, faced high job demands, and suffered from COVID-19 burnout exhibited higher levels of anxiety. By contrast, those who exercised more, maintained desirable family relationships, and perceived strong group cohesion exhibited a lower risk of anxiety. The prevalence of depression was greater in prison officers who were over the age of 30, experienced significant work–family conflicts, faced high job demands, exhibited COVID-19 burnout, and could not tolerate uncertainty regarding COVID-19. Meanwhile, prison officers were less likely to experience depressive symptoms if they earned an annual income of greater than 150,000 RMB, exercised more, maintained desirable family relationships, had a strong sense of job autonomy, and demonstrated effective group cohesion.

This study suggests that a correlation exists between demographic characteristics and the incidence of anxiety and depression among prison officers. Older prison officers report greater anxiety and depression than younger officers. This finding contrasts with previous studies conducted among prison officers, frontline civil servants, and ordinary citizens (7, 11, 61). This discrepancy may be because the prior surveys were performed during the COVID-19 outbreak’s onset, when young people often had to undertake greater emergency work than older people. As the COVID-19 pandemic persisted, older prison officers were confronted with increasing work–family conflict and physical burdens. Therefore, they were more likely to experience anxiety and depression during prolonged closed-loop periods. Moreover, we found that unmarried prison officers were more likely to experience anxiety than their married counterparts. This finding is consistent with a survey of Bangladeshi doctors administered during the COVID-19 pandemic (62). Social support, a sense of stability, and opportunities to express negative emotions afforded by marriage can mitigate the pressures faced by prison officers during the COVID-19 pandemic. In fact, the married population always exhibits better mental health than the unmarried population, and being single is an important risk factor for anxiety (63).

Our analysis revealed that a personal annual income of greater than 150,000 RMB is a protective factor against depression among prison officers. Prior research has demonstrated that mental illness is frequently associated with economic hardship (64, 65), and the risk of depression among people with stable annual income was half that among those with reduced annual income during the COVID-19 pandemic (66). The three-year-long COVID-19 pandemic has exerted a significant negative economic impact. Although prison officers’ personal income has not changed, their family’s annual income may have decreased. High-income prison officers are less likely to be affected by deteriorating economic conditions. Notably, while the chi-square test revealed differences in the mental health of prison officers with different job positions, the binary regression analysis did not identify job position as a risk or protective factor. This is consistent with previous survey findings on the mental health of civil servants and medical teams (11, 67).

Regarding healthy lifestyle, exercise is associated with lower levels of anxiety and depression. Previous research has discovered that during the COVID-19 pandemic, increased exercise could alleviate anxiety and depressive symptoms in college students (68), and inactive individuals were more likely to experience anxiety, depression, and other mental disorders than those who frequently engage in physical exercise (69). The mastery hypothesis posits that during or after exercise, individuals would experience enhanced self-worth, a sense of control over their surroundings, which are crucial for maintaining mental health (70).

Additionally, our study found that prison officers with positive family relationships were less likely to suffer from anxiety and depression, while those experiencing significant work–family conflict exhibited a higher prevalence of anxiety and depressive symptoms. Family functioning significantly impacts mental health (71). Emotional support and regular communication provided by parents or spouses positively affect mental health status (72, 73). In Chinese culture, the importance of family is emphasized, and maintaining close family ties is considered a universal moral requirement (74). Furthermore, Chinese families tend to have closer family connections and provide mutual support to cope with life’s pressures and difficulties (75). However, owing to COVID-19, prison officers were isolated for extended periods and had to live separately from their families, thereby reducing communications with family members and precipitating feelings of loneliness. Previous studies have found that social isolation due to physical restrictions adversely affect mental health (13, 76), and increasing shared feelings with family members mitigates this effect (77). Additionally, a meta-analysis on work–family conflict indicated that when individuals struggle to effectively manage the contradictions between family and work, they may provoke arguments with their family members, consequently diminishing life satisfaction and contributing to mental health problems (78). Therefore, a harmonious family environment is a protective factor that enhances well-being and reduces mental health problems.

Our findings revealed an association between workplace conditions and mental health. From one perspective, heavier workload increases the probability of prison officers experiencing anxiety and depression, which is consistent with studies examining doctors and prison officers during the COVID-19 pandemic (51, 64). The heavy workload of COVID-19 prevention and control increased pressure (79), thus causing mental health problems. By contrast, group cohesion in the work environment protects mental health. Prison officers often lack contact with the external world; thus, they may require greater support from each other than other occupational groups. A previous study found that lack of support from colleagues was related to job burnout among prison officers (80). Finally, positive job autonomy was protective against depression. A previous study revealed that lower professional autonomy and higher job strain are significantly associated with major depressive episodes among nurses (81). According to self-determination theory, low levels of job autonomy are associated with mental health issues in the workplace (82).

Moreover, varying perceptions regarding COVID-19 have contributed to differences in mental health. COVID-19 burnout is a danger sign for anxiety among prison officers; COVID-19 burnout and intolerance of uncertainty regarding COVID-19 are risk factors for depression. Interestingly, this study found no correlation between COVID-19 worry and mental health, which differs from investigations of prison officers in Turkey and the United States (14, 25). This may be because these studies were conducted when people possessed less knowledge regarding COVID-19, and the original strain had a higher fatality rate. When this study was conducted, the ongoing COVID-19 variant was Omicron, and its fatality and toxicity had greatly reduced (83). Additionally, this is attributable to the government’s extremely strict epidemic-prevention measures, which prevented the COVID-19 pandemic from spreading widely in China. Therefore, understandably, people no longer feared or worried about COVID-19. These results indicate that prison officers are not worried about COVID-19 itself but are troubled by the COVID-19 pandemic’s destructive effects on their daily life and work.

At the time of writing, China’s prison system is no longer implementing the “lockdown shifts” strategy. However, these findings not only prepare for potential future closed-loop management scenarios but also provide insights for addressing prison officers’ current mental health concerns. When closed-loop management is again required owing to major public health events in the future, greater attention should be paid to senior and unmarried prison officers. More advanced psychological evaluations can be used, and conducting online psychological counseling for high-risk groups concurrently is highly necessary. Additionally, promoting healthy lifestyles among prison officers, including regular eating habits and exercise, is essential. Designated exercise periods during working hours can be established. Moreover, once the pandemic subsides, prison authorities can permit family visits for officers following nucleic acid testing; enhancing family time can help them cope with negative emotions. Courses on family relationship management can be offered. Furthermore, implementing group counseling activities can strengthen cohesion among prison officers and cultivate a sense of honor and belongingness in their profession. Finally, aiming to alleviate pandemic burnout’s detrimental effects and intolerance of uncertainty regarding virus, it is essential to send positive signal regarding the COVID-19 epidemic to prison officers and help them build confidence to overcome it. To alleviate Chinese prison officers’ current mental health problems, we believe that as their normal life and work routines return to normalcy, their mental health status will improve. However, we still must focus on prison officers who have suffered severe economic losses, broken family relationships, and developed various bad habits under the influence of the three-year epidemic and related policies, as these tangible negative effects may not be eliminated in the short term, even with the relaxation of COVID-19 controls.

## 5. Limitations

This study has four main limitations. First, as a cross-sectional study, it reveals associations but not causality among the studied variables. Additionally, the study only elucidates the prevalence of mental health problems of the prison officers in June and July 2022; however, it cannot capture dynamic changes over time. In the future, our framework can be utilized to collect data longitudinally to assess prison officers’ mental health and identify relevant influencing factors. This approach not only enables causal relationships’ clarification but also provides an understanding of the mental health changes within this group as public health events evolve. Second, this study only relied on the prison officers’ self-reported data; participants may have provided socially desirable responses that do not reflect their actual status. Future research can incorporate evaluations from both family members and colleagues to comprehensively assess prison officers’ mental health status. Third, this study found that annual income ≥150,000 RMB, job autonomy, and intolerance of uncertainty regarding COVID-19 only influenced prison officers’ depression but not anxiety. The reasons and underlying mechanisms behind this interesting result can be further explored in future studies. Fourth, our data were limited to the west and south of China. Different regions may have varying levels of control over the COVID-19 pandemic; future investigation can consider this factor for further analysis. Moreover, the lockdown shift strategy’s details in different prisons are also inconsistent, and the mental health of prison officers from other districts also merits consideration. Therefore, examining the mental health status of prison officers from various districts in China is necessary, especially if the prison system implements a closure policy again in the future.

## 6. Conclusion

This study is the first to examine the prevalence of anxiety and depression among prison officers after being affected by the prolonged COVID-19 pandemic. Our findings revealed a significantly high prevalence of depression and anxiety compared to the outbreak’s early stage; the high prevalence associated with sociodemographic characteristics, family environment, healthy lifestyles, workplace conditions, and perceptions of COVID-19. This study offers a guide for the prison system to enhance prison officers’ mental health status during future closed-loop management periods.

## Data availability statement

The raw data supporting the conclusions of this article will be made available by the authors, without undue reservation.

## Ethics statement

The studies involving human participants were reviewed and approved by Ethics Committee of the School of Sociology at the China University of Political Science and Law. The patients/participants provided their written informed consent to participate in this study.

## Author contributions

XL, YZ, and JX conceived the idea and analyzed the data. YZ, QZ, and JX conducted the investigation and drafted the initial manuscript. JX and YZ were responsible for data curation. XL reviewed and edited the manuscript. AM and XL supervised the project and secured funding. All authors contributed to the article and approved the submitted version.

## Funding

This study received support from the “Scientific Research Innovation Project of China University of Political Science and Law” (the Fundamental Research Funds for the Central Universities) awarded to AM (nos. 20ZFD82002; ZFYZ88001); the “Humanities and Social Sciences Fund of the Chinese Ministry of Education” provided to AM (no. 21YJAZH058); the Beijing Social Science Fund provided to AM (no. 21JYB010); the major project of the Chinese Ministry of Education, National Education Sciences Planning Fund awarded to XL (no. DEA220353).

## Acknowledgments

The authors extend their appreciation to the participants who completed the questionnaires and the prison officers who facilitated the execution of this research.

## Conflict of interest

The authors declare that the research was conducted in the absence of any commercial or financial relationships that could be construed as a potential conflict of interest.

## Publisher’s note

All claims expressed in this article are solely those of the authors and do not necessarily represent those of their affiliated organizations, or those of the publisher, the editors and the reviewers. Any product that may be evaluated in this article, or claim that may be made by its manufacturer, is not guaranteed or endorsed by the publisher.

## Footnotes

^1^ www.wjx.cn
